# Sleeping Beauty and the Microenvironment Enchantment: Microenvironmental Regulation of the Proliferation-Quiescence Decision in Normal Tissues and in Cancer Development

**DOI:** 10.3389/fcell.2018.00059

**Published:** 2018-06-07

**Authors:** Ana Paula Zen Petisco Fiore, Pedro de Freitas Ribeiro, Alexandre Bruni-Cardoso

**Affiliations:** e-Signal Laboratory, Departamento de Bioquímica, Instituto de Química, Universidade de São Paulo, São Paulo, Brazil

**Keywords:** quiescence, proliferation, growth, microenvironment, extracellular matrix, tissue architecture, cancer, cell cycle

## Abstract

Cells from prokaryota to the more complex metazoans cease proliferating at some point in their lives and enter a reversible, proliferative-dormant state termed quiescence. The appearance of quiescence in the course of evolution was essential to the acquisition of multicellular specialization and compartmentalization and is also a central aspect of tissue function and homeostasis. But what makes a cell cease proliferating even in the presence of nutrients, growth factors, and mitogens? And what makes some cells “wake up” when they should not, as is the case in cancer? Here, we summarize and discuss evidence showing how microenvironmental cues such as those originating from metabolism, extracellular matrix (ECM) composition and arrangement, neighboring cells and tissue architecture control the cellular proliferation-quiescence decision, and how this complex regulation is corrupted in cancer.

## Introduction

Proliferation is one of the most conserved and fundamental traits of cells. However, all cells from prokaryota to mammals stop proliferating at some point during their lifetime in a controlled and reversible process called quiescence (O'Farrell, 2011). But why did quiescence triumphed during evolution and how is it controlled? For a number of unicellular organisms, the answer is very straightforward: environmental limitations. These limitations, mainly in the form of nutrient scarcity, acted as selective pressures that favored the success of those unicellular organisms that could quit proliferation and then “wake up” later on when the conditions were more suitable.

The gift of “sleeping” and thus remaining viable in adverse situations was vital to the perpetuation of genetic material and the success and ubiquity of unicellular organisms and also to the appearance of their multicellular descendants. While several key traits of unicellular quiescence (e.g., core signaling pathways, survivability, and reversibility) remain in higher organisms, in more complex living systems, such as mammals in which most cells are found to be quiescent, (macro)environmental pressures do not seem to contribute to quiescence. Cells become quiescent even in the presence of abundant resources (Spencer et al., 2011, 2013; Valcourt et al., 2012; Fiore et al., 2017).

Under the optimal conditions of nutrients, growth factors, and mitogens, cells “know” when to proliferate, but they need to be “told” when to stop. In other words, although proliferation might be considered a default setting (Parr, 2012), cells retain a built-in program of quiescence, which is set off extrinsically. This ability to become proliferative-dormant is essential to the acquisition of the function, defined geometry, and size of complex organs such as the heart, kidney, brain, and mammary glands. In these organs, a cell resides in a milieu composed of extracellular matrix (ECM) molecules, soluble factors, and other cells that produce various chemical and physical signals. This milieu is termed as the microenvironment, and it influences all aspects of a cell's life. It is the microenvironment that “tells” the cell to stop proliferating and activates the quiescence program.

Changes in the microenvironment too can “wake up” cells in a controllable fashion; as is the case in situations where cells need to proliferate to perform their functions (e.g., dermal fibroblasts upon wound healing and lymphocytes during the immune response) or to compensate for cell loss (e.g., intestinal epithelial cells and epidermal cells). Of course, the cell itself can also influence its microenvironment. A cell can instruct and modify its microenvironment by remodeling the ECM, and physically and chemically networking with its neighboring cells. Nevertheless, the reciprocal exchange between the cell and its microenvironment (Bissell et al., 1982), that finely regulates all aspects of cell behavior and fate including quiescence, is disrupted in cancer. The microenvironment “enchantment” is lost and cells “wake up” to resume proliferation, but this time in an unrestrained manner.

Here, we consider molecular aspects of cell cycle regulation and discuss how the cooperation of different microenvironmental signals are critical to the proliferation-quiescence decision, and how this orchestrated regulation goes awry in cancer. Because cellular quiescence programs are actively triggered by tissue- and conditional—specific factors, we argue that uncontrolled cell division, seen frequently in cancer, does not solely depend on oncogenic activation of the cell cycle, but also occurs due to loss of control over quiescence imposed by the microenvironment.

## Cell cycle regulation and definition of cellular quiescence

**Figure 1:**
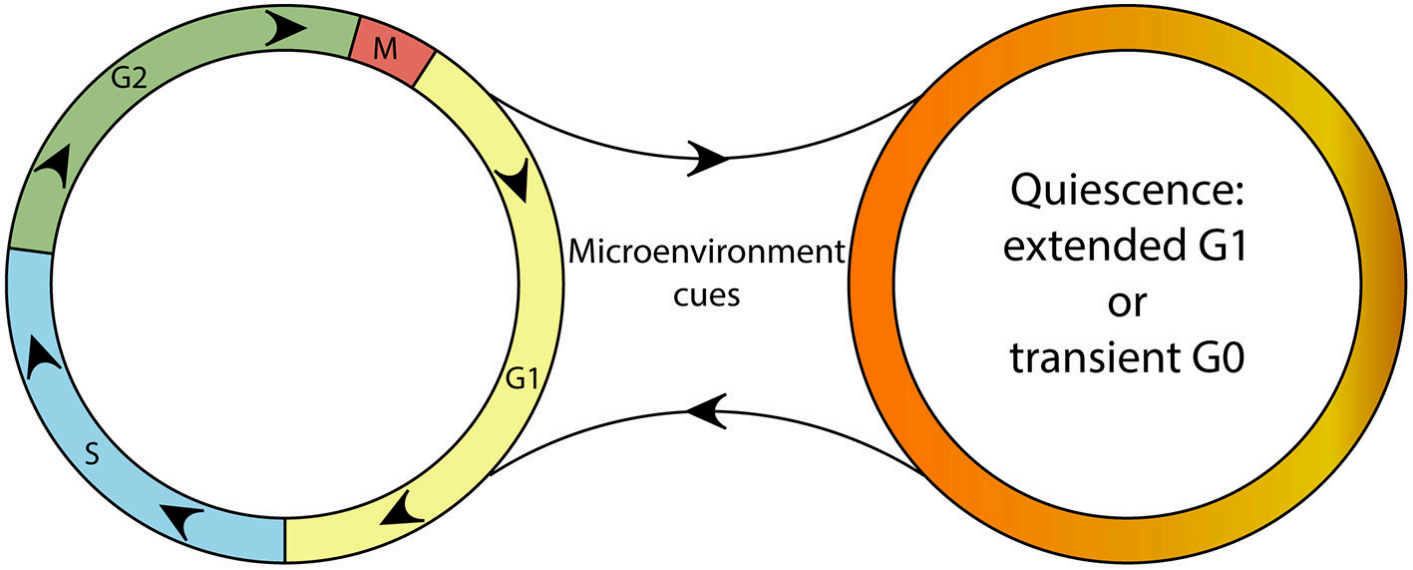
A schematic depicting the continuity of the cell cycle and the quiescence compartment. Although a continuous cell cycle resulting in proliferation might be considered the “default program” of cells, microenvironmental cues can trigger the “optional setting” of quiescence. Quiescent cells are able reenter the cell cycle upon changes in their microenvironment.

Proliferation is one of the most conserved and elemental attribute of a living system. Because of its importance for reproduction, tissue growth, and regeneration, and also its status as a hallmark of cancer, a lot is known about the molecular mechanisms that control cell proliferation. To divide, cells must go through a series of necessary steps termed the cell cycle. The cell cycle is divided into four phases characterized by a set of discrete events: growth and preparation for DNA replication (G1), DNA replication (S), preparation for mitosis (G2), and mitosis (M) and culminates with cell division (Figure 1). Although a large number of signals can trigger cell cycle entry and their molecular details may vary, key elements of the cell cycle are extremely well-conserved from yeast to mammals (Harashima et al., 2013). For instance, as a rule, the cell cycle is triggered by growth-factor signaling pathways that activate cyclin-dependent kinases (CDKs) which in turn are cyclically activated and mainly regulated by another class of proteins called cyclins (Bloom and Cross, 2007). The content of cyclins is mainly controlled at the gene expression level and post-translationally by degradation via proteasome (Bloom and Cross, 2007; Harashima et al., 2013). Another layer of regulation is provided by the two groups of cyclin-CDK inhibitors, the INK4 inhibitors (p15, p16, p18, p19), and the kip/Cip proteins (p21, p27, p57) (Polyak et al., 1994a,b; Pagano et al., 1995; Sherr and Roberts, 1999), and by the retinoblastoma protein (pRb). pRb represses the transcription factor E2F, which initiates the transcription of cell cycle activators. CDK-mediated hyperphosphorylation of pRb releases E2F promoting cell cycle progression (Fischer and Muller, 2017).

Unlike proliferation, little is known about the molecular biology of cellular quiescence. While proliferating cells from different tissues share many similarities, quiescent cells differ a lot in their expression programs (Bissell, 1981; Coller et al., 2006; Coller, 2011). There exist several types of quiescence, differing from organism to organism, cell to cell, and at different times and locations within an organ (O'Farrell, 2011). Few studies have focused on defining the molecular markers and/or signatures of different types of quiescence induction (Johnson et al., 1975; Williams and Penman, 1975; Schneider et al., 1988; Gos et al., 2005; Coller et al., 2006; Liu et al., 2007; Coller, 2011). In general, quiescent cells possess a transcriptional profile different from cycling cells, achieved by downregulating proliferation and cell-cycle progression genes, and upregulating genes that are not only related to cell cycle inhibition but also give the cells new properties (Coller et al., 2006; Coller, 2011; O'Farrell, 2011). Furthermore, depending on the type of inhibition of proliferation, the group of upregulated genes in quiescence can vary considerably (Coller et al., 2006).

Given the non-uniform nature of cellular quiescence (Coller et al., 2006; O'Farrell, 2011) and hence, the lack of a universal marker and/or genetic signature thereof, the assessment and definition of quiescence pose a difficult task and is subject to ongoing debate. To determine quiescence, some researchers have relied on the expression of CDK inhibitors like p27^kip1^, which are usually elevated in quiescent cells (Polyak et al., 1994a,b; Coller, 2011). However, high levels of CDK inhibitors are also associated with entry into senescence and terminal differentiation (Ruas and Peters, 1998; Sherr and Roberts, 1999; Bringold and Serrano, 2000). Additionally, overexpression of CDK inhibitors does not reproduce the transcriptional signature of quiescent cells (Coller et al., 2006; Sang et al., 2008). It is also disputed whether cells in quiescence enter a non-cycling “compartment” termed G0 or halt in G1 (Smith and Martin, 1973; Shields and Smith, 1977; Spencer et al., 2013; Arora et al., 2017; So and Cheung, 2018). Although presenting the same amount of non-replicated DNA, quiescent cells spend longer periods of time between mitosis exit and S phase and express different sets of genes, when compared to active cycling cells in a canonical G1 (Coller et al., 2006; Sang et al., 2008; Coller, 2011; Spencer et al., 2013; Arora et al., 2017).

We support the idea that the best way to identify quiescent cells relies on properties not detected in cycling cells and absence of proliferative traits such as cell cycle activating factors, DNA synthesis, and mitotic markers (O'Farrell, 2011). Also, there is some confusion when distinguishing quiescence from replicative senescence. Senescent cells are found in an essentially permanent growth arrest induced by extrinsic and genotoxic stresses (Campisi and d'Adda di Fagagna, 2007; Rodier and Campisi, 2011) and express markers not found in quiescent cells like senescence-associated β-galactosidase, nuclear foci containing DNA damage proteins (DNA-SCARS) and senescence-associated heterochromatin foci (SAHF) (Rodier and Campisi, 2011). Quiescent cells are those uncommitted to any proliferation-related activity but are also not in irreversible states such as senescence, terminal differentiation, or apoptosis. While the only other option for senescent and terminally differentiated cells is cell death, quiescent cells are capable of many cell fates such as reverting to proliferation, differentiating, senescing, or dying.

The ability of becoming quiescent is found in many different cells types and conditions, including non-malignant, malignant, undifferentiated, and differentiated cells from distinct tissues in normal and aberrant situations. For example, differentiated hepatocytes in a normal liver have the capacity to reenter the cell cycle upon physical (i.e., resection) and chemical injury (Kim et al., 1997; Michalopoulos and DeFrances, 1997; Presnell et al., 1997; Dong et al., 2007); Quiescence is also crucial for maintenance of adult stem cells (Wilson et al., 2008; Fu et al., 2017). Stem cell microenvironmental cues can either trigger quiescence or direct stem cells toward proliferation and functional differentiation (Cheung and Rando, 2013). In cancer dormancy, residual cancer cells disseminated from the primary tumors survive in a quiescent state in distant organs. These dormant cells appear to be responsible for metastases that occur years or even decades after tumor surgery and treatment (Ghajar et al., 2013; Sosa et al., 2014).

Cellular quiescence was defined in the past as a default state of inactivity (Cheung and Rando, 2013) acquired passively when conditions are not optimal for proliferation. But, a growing body of compelling evidence compiled herein shows that quiescence is a molecularly diverse, non-terminal and tissue-specific state that can be activated and sustained by the cell microenvironment.

## Changes in systemic, tissue and cell metabolism regulate the proliferation-quiescence decision

In metazoans, in addition to favorable nutritional conditions, ligands like growth factors, mitogens, and conditional signals are necessary to trigger a receptor response to promote and regulate growth and proliferation (Valcourt et al., 2012). Core cell growth pathways like PI3Kinase/AKT/TOR are conserved from yeast (a unicellular eukaryote) to Metazoa and connect biochemical cues and nutrient availability with the cell cycle re-entry of quiescent cells (Vivanco and Sawyers, 2002). In yeast, growth pathways are not coupled to extracellular signaling molecules. Rather, they proliferate if provided with sufficient nutrients and appropriate pH, temperature, and pressure (Soto et al., 2016).

Quiescence signaling in animal cells can be triggered even when conditions are suitable to sustain proliferation, such as with sufficient nutrients and growth factors (Weaver et al., 1997; Wang et al., 1998; Liu et al., 2004; Spencer et al., 2013). For example, during formation of 3D acinar structures in a reconstituted basement membrane, cells cease proliferating despite the culture media containing and being regularly replenished with all the additives required for cell growth and proliferation (Weaver et al., 1997; Wang et al., 1998; Liu et al., 2004). Most likely, quiescence in complex organisms is not a consequence of the absence of growth stimuli, but instead an active process of growth suppression (Parr, 2012).

Quiescent cells preferentially oxidize carbon compounds to produce ATP to drive basic cellular processes. Proliferating cells, however, shift their metabolism to more anabolic pathways so they can generate biomass and commit to cell division (Vander Heiden et al., 2009; Palm and Thompson, 2017). The first metabolic signature associated with cancer was that tumors take up glucose and break it down into lactate more quickly than normal tissues, even in the presence of an abundance of oxygen that would otherwise lead to the complete oxidation of glucose in the mitochondria (Rabinowitz and Coller, 2016). This phenomenon is called the Warburg Effect, named after Otto Warburg, a biochemist who first observed the phenomenon by comparing lactate production in normal and tumor tissues (Kim and Dang, 2006; Koppenol et al., 2011). Proteins downstream of growth factor signaling regulate metabolism and display oncogenic potential. Pathways centered on PI3K-AKT and RAS proteins, for instance, are critically involved in the abnormal metabolism of cancer cells, including the Warburg effect, enhanced proliferation, and survival (Thompson, 2009; Pavlova and Thompson, 2016). On the other hand, tumor suppressors, like PTEN, a PI3K signaling inhibitor, and p53 can oppose unrestrained proliferation, and therefore, mutations in these genes also contribute to abnormal metabolism (Thompson, 2009).

Altered metabolic routes, including not only that of carbohydrates but also amino acid and lipid metabolism, are a hallmark of cancer and are essential to sustain the uncontrolled proliferation and survival of tumor cells (Hirschey et al., 2015; Coloff et al., 2016; Keckesova et al., 2017) (Hanahan and Weinberg, 2000, 2011). For example, proliferating cells metabolize significantly more glutamate via transaminases, whereas quiescent cells consume less glutamine and have reduced non-essential amino acid (NEAA) synthesis (Coloff et al., 2016). Highly proliferative tumors couple glutamine usage to NEAA production to sustain biosynthesis (Coloff et al., 2016). The Weinberg lab (Keckesova et al., 2017) performed transcriptome analysis of experimentally induced quiescent and differentiated muscle cells to identify that Lactamase B (LACTB), a mitochondrial protein, was upregulated in relation to undifferentiated and cycling cells. LACTB was able to strongly inhibit proliferation in multiple breast cancer cell lines by changing mitochondrial lipid metabolism and reducing the levels of mitochondrial phosphatidylserine decarboxylase (Keckesova et al., 2017).

**Figure 2:**
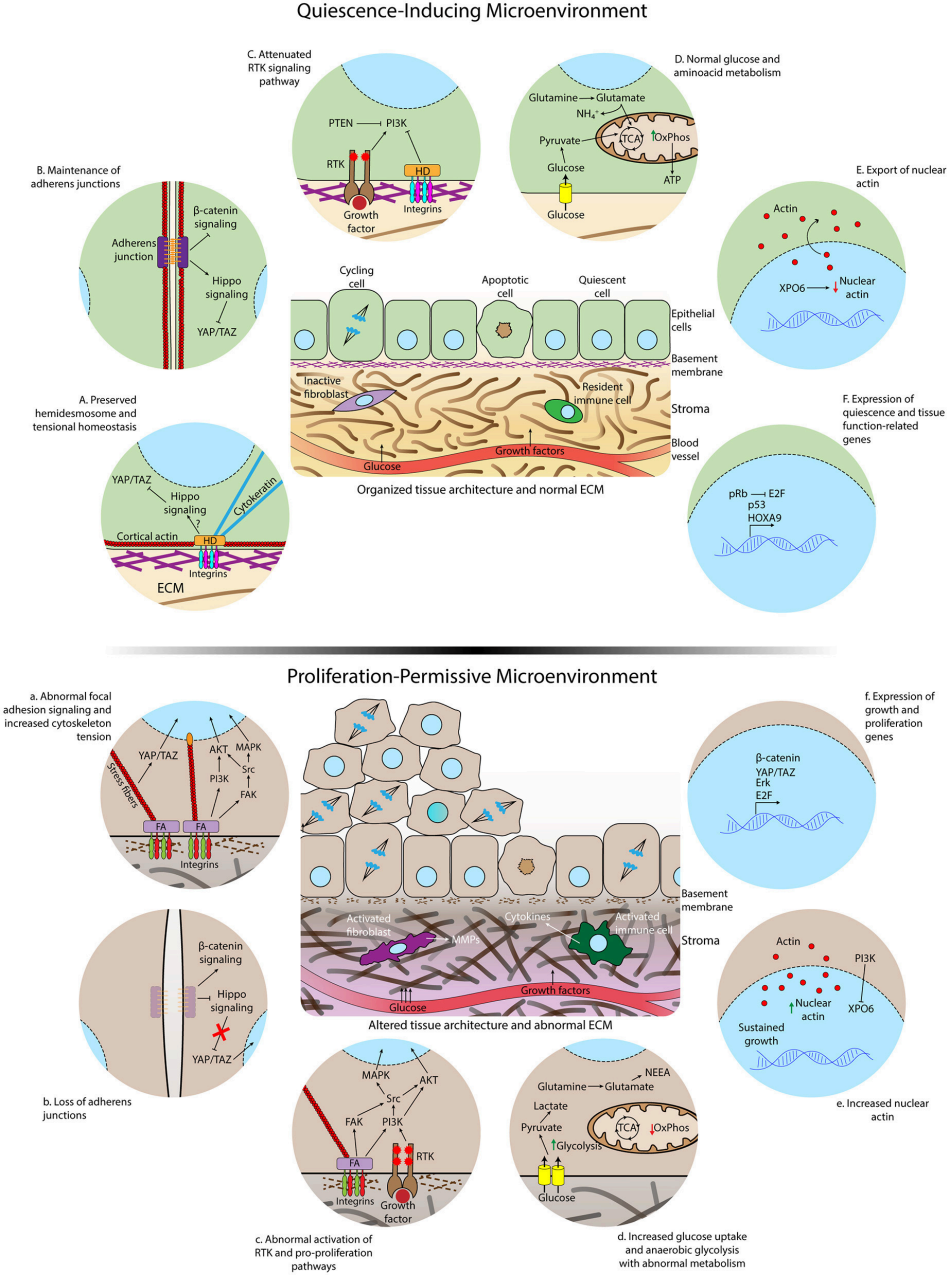
Differences in microenvironmental and subcellular signaling between homeostatic conditions and loss of cell quiescence. **(Top)** Quiescence-inducing microenvironment showing normal extracellular matrix, intact basement membrane, organized tissue architecture, and resident immune cells and fibroblasts. The rates of proliferation and apoptosis are comparable to maintain homeostasis. **(Below)** Altered and proliferation-permissive microenvironment displaying abnormal extracellular matrix with altered composition and structure, disrupted basal lamina, activated fibroblasts in the stroma, inflammatory infiltrate by activated macrophages and other cytokines-secreting activated immune cells. The rate of proliferation is increased due to loss of quiescence regulation by the tissue microenvironment. Details of epithelial cells residing in a healthy **(A-F)** or aberrant microenvironment **(a–f)**. A normal ECM and correct tissue architecture induces the formation of hemidesmosomes connecting the ECM to cytokeratin filaments, cell-cell junctions, cortical actin cytoskeleton, and polarized epithelium **(A,B)**. Consequently, the Hippo pathway, that inhibits translocation of YAP/TAZ to the nucleus, is activated, receptor tyrosine kinase (RTK) activity is attenuated **(C)** and nuclear actin export is enhanced **(E)**. Glucose and glutamine are completely metabolized by the tricarboxylic acid cycle (TCA) and oxidative phosphorylation (OxPhos) **(D)**. Quiescence gene expression programs are triggered during quiescence acquisition **(F)**. Aberrant ECM signaling due to altered ECM composition and stiffness increases formation of focal adhesions (FA), loss of cortical distribution of the actin cytoskeleton, enhanced activation of FAK, formation of actomyosin stress fibers, translocation of YAP to the nucleus **(a)** and accumulation of nuclear actin **(e)**. Loss of adherens junctions allows the translocation of beta–catenin to the nucleus **(b)**. Overactivation of growth factor signaling occurs as a consequence of intersection between integrin and RTK-triggered signaling **(c)**. Glucose uptake and anaerobic glycolysis are further increased due to exacerbated activation growth factor pathways, glutamine is converted to NEAA and biosynthetic precursors in the TCA and oxidative phosphorylation is reduced in the aberrant microenvironment **(d)**. Activation of genes involved in cell-cycle entry is increased **(f)**.

The metabolic shift displayed by tumors was first considered exclusively as a consequence of cancer cell-autonomous processes such as mutations in proto-oncogenes and tumor suppressors that are primarily involved in growth signaling and cell cycle regulation. However, growing epidemiological and experimental evidence has shed light on the role of disrupted metabolism in oncogenesis (Onodera et al., 2014; Hirschey et al., 2015). Metabolic diseases such as type II diabetes and obesity correlate with an enhanced risk of developing many types of cancer (Giovannucci et al., 2010; Doerstling et al., 2017). The molecular mechanisms behind this correlation are still unclear and may be linked to aberrant growth factor-stimulated signaling, hyperglycemia, chronic inflammation associated with obesity and diabetes or indeed a combination of these processes (Giovannucci et al., 2010). Experimentally, Onodera et al. showed that increased glucose uptake and glycolysis can elicit malignant phenotypes in non-malignant breast cells, including activation of oncogenic pathways like EGFR, β1-integrin, PI3K, and MAPK, loss of tissue polarity and importantly, loss of quiescence. Reducing glucose uptake in malignant cells, conversely, resulted in gain of polarity and cell cycle arrest (Onodera et al., 2014). This may help explain why metformin, a drug used to treat type II diabetes, reduces the incidence of cancer in diabetes patients and is regarded as a potential treatment for some types of cancer (Ben Sahra et al., 2011; Loubière et al., 2013; Rosilio et al., 2014; Higurashi et al., 2016). The experimental data together with epidemiological evidence indicate that a hyperglycemic microenvironment may contribute to exit from quiescence of already genetically altered cells in the early stages of tumorigenesis and the consequent development of neoplastic lesions (Figure 2).

## The composition and physical properties of the extracellular matrix are key determinants of cellular quiescence

The ECM is composed of a complex network of biochemically diverse molecules including glycoproteins, non-glycosylated proteins, proteoglycans, and polysaccharides that assemble into three-dimensional scaffolds with a myriad of biochemical and biomechanical properties. By acting as ligands of cell surface receptors like integrins, the ECM molecules not only make the structural frame that determines the morphology and physical properties of tissues and organs but are also crucial in tuning cell signaling pathways, including growth factor-stimulated pathways, that control all aspects of cell behavior (Hynes, 2009; Lu et al., 2011, 2012; Pickup et al., 2014). Cell to ECM adhesion mediated by heterodimers of integrins is required for cell proliferation. Responses to growth factors depend on the cell being anchored via integrins to a component of the ECM (Schwartz and Assoian, 2001; Hynes, 2002; Ivaska and Heino, 2011; Pickup et al., 2014). For example, insulin stimulation promotes binding of the integrin pair αvβ3-integrin with insulin receptor substrate-1 (IRS-1) and this linkage is necessary to induce DNA synthesis (Vuori and Ruoslahti, 1994). Blocking β1-integrin binding to ECM inhibited CDK activity leading to an accumulation of hypophosphorylated pRb (Day et al., 1997). Furthermore, there are numerous studies showing that intracellular signaling induced by integrin-binding to the ECM are analogous to, and intersect with, growth-factor pathways like RAS-MAPK and PI3K-AKT (Yu et al., 2001; Cabodi et al., 2004, 2010; Ivaska and Heino, 2011). Notably, the ECM also functions as a “reservoir” of a large number of growth factors and cytokines. The ECM can, in this way, store molecules that can be released by proteases and can induce proliferation in specific locations (Bonnans et al., 2014).

The ECM is divided into two main components: the interstitial matrix and the basement membrane. The interstitial matrix contains fibrillar collagens and different types of proteoglycans and glycoproteins made mostly by stromal cells (Figure 2). The basement membrane (BM) is a specialized thin layer of ECM produced by both epithelial cells and stromal cells composed almost exclusively of laminins, type IV collagen, nidogen, and proteoglycans (Lu et al., 2012). Besides separating the epithelial and stromal compartments, the BM plays key roles in instructing the epithelial cells to differentiate, function, and survive. For example, laminin-rich ECM (lrECM), a surrogate for the basement membrane, induces tissue polarization and quiescence and also reprograms gene expression in mammary gland epithelial cells (Barcellos-Hoff et al., 1989; Streuli and Bissell, 1991; Boudreau et al., 1995, 1996; Streuli et al., 1995; Petersen et al., 1998; Kenny and Bissell, 2003; Akhtar et al., 2009; Spencer et al., 2011; Fiore et al., 2017). Additionally, the basement membrane suppresses apoptosis of mammary epithelial cells (Boudreau et al., 1995). These findings support the notion that quiescent cells not only cease proliferating but also undergo genetic reprogramming acquiring new properties (Coller et al., 2006; Coller, 2011; O'Farrell, 2011) and that quiescence programs might overlap with survival signaling resulting in resistance to cell death.

Normal epithelial cell lines grown in 3D laminin-111 rich gels have been shown to form quiescent acinar structures with polarized cells. In contrast, malignant cells grow as disordered proliferating structures (Rizki et al., 2008; Fiore et al., 2017; Wessels et al., 2017). Laminin-111 levels are reduced and show irregular distributions in several stages of breast cancer (Petersen et al., 1992). Increased expression and aberrant deposition of collagen I and IV and fibronectin induced proliferation, loss of polarity, and a malignant phenotype in several types of epithelial cells (Hoffman et al., 1996; Zhang et al., 2002; Wozniak et al., 2003; Provenzano et al., 2009; Malik et al., 2010; Schedin and Keely, 2011; Espinosa Neira and Salazar, 2012; Kim and Gumbiner, 2015). In non-malignant cells from the mammary gland, signals from laminin-111 increase the levels of nitric oxide and active p53 (Furuta et al., 2018) and decrease the level of nuclear actin via export by exportin-6 (XPO6) (Fiore et al., 2017), resulting in polarized and quiescent acini. Actin in the nucleus is essential for RNA synthesis and RNA levels (Percipalle, 2013; Virtanen and Vartiainen, 2017) which, in turn, are crucial to sustain cell proliferation (Spencer et al., 2011; Fiore et al., 2017). In malignant cells; XPO6 activity is not enhanced, nuclear actin and RNA levels are not decreased, and malignant cells are unresponsive to quiescence-inducing cues from laminin-111 (Fiore et al., 2017). Interestingly, inhibition of PI3K results in increased N-actin export (Fiore et al., 2017). Although quiescence involves genetic reprogramming leading to the acquisition of new properties (Coller et al., 2006; Coller, 2011), it is still uncertain whether the cooperation of laminin-111 signaling and nuclear actin influences the functional differentiation of the mammary gland. In addition, it is unclear which configuration of nuclear actin, either monomeric or filamentous, regulates cell proliferation and quiescence.

Cancer cells have the ability to remodel their own ECM by secreting pro-proliferation ECM components like fibronectin (Hynes and Naba, 2012; Naba et al., 2014, 2017). The nature of the ECM receptors bound to the cancer cell-secreted ECM may enhance proliferation-promoting pathways and/or attenuation of growth inhibitory signals. Additionally, active degradation of laminin-111 and the consequent breakdown of the supramolecular structure of the BM by matrix metalloproteinases (MMPs), enzymes that are commonly overexpressed in different stages of tumor development of several cancers (Kessenbrock et al., 2010; Bonnans et al., 2014), result in re-activation of the cell cycle (Beliveau et al., 2010) leading to subsequent development of tumors (Bissell et al., 2005; Calvo et al., 2013). Importantly, key genetic alterations (e.g., oncogene activation and loss of tumor suppressors) commonly found in malignant cells induce changes in the microenvironment and on how cells sense their surroundings. For instance, BRAF^V600E^ mutation induces expression of both α3/α6 integrins (Nucera et al., 2010) and thrombospondin-1 (*THBPS1*) in thyroid papillary carcinoma, as well as in melanome, eliciting hyperproliferation and cell invasion (Nucera et al., 2010; Jayachandran et al., 2014).

Despite carrying several genetic alterations, disseminated tumor cells (DTCs) can survive in a reversible dormant state for decades in secondary sites (Sosa et al., 2014). Strong evidence from mouse and co-culture experiments has shown that the ECM of microvascular niches is crucial to influence the proliferation-quiescence decision in breast DTCs. Thrombospondin-1 in the BM of mature endothelial cells sustained quiescence, whereas sprouting neovasculature is rich in active TGF-β1 and periostin induced proliferation of breast cancer cells (Ghajar et al., 2013).

Epithelial cells in normal and neoplastic tissues reside in a cell-rich microenvironment containing resident fibroblasts, endothelial cells, pericytes, and immune cells. These cells have active roles in the tissue microenvironment biology, especially in ECM remodeling. Cancer-associated fibroblasts (CAF) and tumor-associated macrophages (TAM) are abundant in the tumor microenvironment playing major roles in remodeling the ECM and establishing paracrine signaling with neoplastic cells that support cancer development (Kalluri, 2016). CAFs derived from human prostate tumors were able to induce malignant transformation and proliferation of non-malignant, but genetically initiated, prostate epithelial cells (Olumi et al., 1999). Human mammary epithelial cells transfected with SV40 large-T antigen, the telomerase catalytic subunit, and an H-Ras oncoprotein when mixed with Matrigel (a commercial ECM) or primary human mammary fibroblasts displayed increased capacity to form tumors in immunocompromised mice (Elenbaas et al., 2001). In part, CAFs communicate with cancer cells via secretion of cytokines and exosomes, small vesicles that contain proteins, nucleic acids, and metabolites that can modulate the behavior of cancer cells (Hoshino et al., 2015; Kalluri, 2016; Zhao et al., 2016; Matei et al., 2017) (Figure 2). Macrophages are key players in chronic inflammation associated with oncogenesis and tumor progression (Balkwill et al., 2005). Noteworthy, chronic inflammation triggered by infectious or chemical agents and tissue-intrinsic mechanisms increase the risk of cancer, and many neoplasms are believed to initiate at sites of chronic inflammation (Balkwill et al., 2005; Karin and Greten, 2005; Kenny et al., 2007; Tyan et al., 2011). TAMs remodel the ECM via secretion of MMPs and promote cell cycle reactivation by producing growth factors and cytokines (Balkwill et al., 2005; Goswami et al., 2005; Deryugina and Quigley, 2015; Vinnakota et al., 2017) (Figure 2).

The ECM is not only a bystander in the tumor microenvironment (Pietras and Ostman, 2010) but rather actively contributes to cancer initiation and progression, and this is an accepted fact. Indeed, it has been suggested that the ECM should be seen as a modulator of all hallmarks of cancer (Pickup et al., 2014) and we argue that an aberrant ECM should also be considered a hallmark of cancer. Similar to the biochemical traits of the ECM, biomechanical cues, specifically ECM stiffness and topology, are also sensed by cells, triggering signal transduction cascades affecting many aspects of cell behavior. Tumor stroma is typically more rigid than normal stroma due to increased collagen deposition and crosslinking between collagen fibers and other ECM molecules catalyzed by abnormal activities of the enzyme lysil oxidase (LOX) (Levental et al., 2009; Lu et al., 2012). Women with dense tissue in 75% or more of the breast have an elevated risk of developing breast cancer, in comparison with women with little or no dense tissue (Boyd et al., 2007). Moreover, patients with pancreatic ductal adenocarcinoma display intense desmoplasia, a process that involves a considerable increase in collagen types I and V, myofibroblastic pancreatic stellate cells, and immune cells, which are associated with cancer progression and poor survival (Pandol et al., 2009). In addition, leiomyomas, a common type of benign tumor, are characterized by neoplastic growth and excessive collagen I and III deposition (Wolanska et al., 1998).

How then does microenvironmental stiffness influence the proliferation-quiescence decision? Focal adhesions (FAs), intracellular protein complexes of protein kinases, and adaptors linked to filamentous actin (F-actin) are formed when cell surface receptors bind ECM molecules (Howlett et al., 1995; Giancotti and Ruoslahti, 1999; Zhao and Guan, 2011; Hynes and Naba, 2012). These complexes are modulated by substrate rigidity in response to intracellular tension built and stored in the actomyosin cytoskeleton (Wozniak et al., 2004; Halder et al., 2012). The critical proteins in FAs are the heterodimers of integrins (usually containing β1-integrin chains) and FAK (Focal Adhesion Kinase). FAK phosphorylates multiple substrates and helps integrate integrin and growth factor signaling pathways (Cabodi et al., 2010). In addition to growth factor-responsive receptor tyrosine kinases, PI3K is activated by other kinases, such as FAK and integrin-linked kinase (ILK), which also relay signals from the ECM (Wang et al., 1998; Grant et al., 2002; Reif et al., 2003; You et al., 2015). Aberrant β1-integrin signaling and increased expression and activity of FAK are frequently associated with tumorigenesis (Desgrosellier and Cheresh, 2010; Zhao and Guan, 2011) and inhibition of either EGFR or β1-integrin can induce the formation of quiescent acinar structures in malignant breast cells (Weaver et al., 1997; Wang et al., 1998; Nisticò et al., 2014). Furthermore, FAK phosphorylation activates pro-proliferative pathways like Src-RAS-MAPK (Zhang et al., 2002; Provenzano et al., 2009). Cell cycle progression requires integrin binding to the ECM that promotes activation of RAS and ERK signaling, re-initiating the cell cycle (Schwartz and Assoian, 2001; Pickup et al., 2014). In response to increased ECM rigidity, cells increase FAK activation that activates cell cycle progression via RAC stimulation (Bae et al., 2014; Pickup et al., 2014). In addition, the activation of FAK promotes nuclear localization of YAP, an effector of the Hippo pathway, through activation of SRC-PI3K signaling (Kim and Gumbiner, 2015). Conversely, inhibition of β1 integrin signaling with the AIIB2 blocking antibody inhibits FAK activation, reverting breast epithelial tumor cells to a normal-like phenotype reassembling the basement membrane and reestablishing cell to cell junctions resulting in decreased cyclin D1 and upregulation of p21 (Weaver et al., 1997; Wang et al., 1998).

In general, these data demonstrate that alterations in the composition of the extracellular matrix may be an important trigger for the activation of FAK-mediated pro-proliferative pathways, thus promoting a mechanism of escape from quiescent programs. Indeed, in leiomyoma cells, the interplay between collagen signaling and the growth factor-stimulated MAPK pathway regulates cell cycle progression (Koohestani et al., 2013). In mammary epithelial cells, a stiff ECM promotes malignant phenotypes by inducing miR-18a, which decreases levels of the transcription factor HOXA9 and the tumor suppressor PTEN, resulting, respectively, in cell proliferation (Gilbert et al., 2010) and enhanced PI3K activity (Mouw et al., 2014) (Figure 2).

While increased FA formation and FAK activity correlate with increased deposition and crosslinking of collagen and aberrant cell-growth pathways, hemidesmosomes seem to counteract these effects on physiological stiffness and are linked with a cell cycle arrest phenotype (Weaver et al., 2002; Chaudhuri et al., 2014; Nisticò et al., 2014). Hemidesmosomes are multiprotein structures mediating cell-ECM adhesion. They possess the integrin pair α6β4 at their core and connect the BM to cytokeratin filaments (Walko et al., 2015). Perturbation of hemidesmosomes is involved in the development and progression of certain cancers (Rabinovitz and Mercurio, 1996; Nisticò et al., 2014). Remarkably, although ECM stiffness induces malignant phenotypic transformation, including escape from cellular quiescence (Paszek et al., 2005; Chaudhuri et al., 2014), this effect is lost when combined with an increase in available basement-membrane ligands (Chaudhuri et al., 2014). Thus, ECM rigidity and composition seem to act in conjunction with one another to regulate malignant phenotypes. This line of evidence indicates that altered physical properties of the ECM and the ECM composition itself should be jointly considered when evaluating the risk of development of hyperproliferative lesions and cancer prognosis (Branco da Cunha et al., 2014; Chaudhuri et al., 2014). Moreover, depending on the biological context, the composition and structural properties of the ECM may drive cell cycle progression as is the case in tissues possessing high densities of collagen fibers. Conversely laminin-111 together with an intact BM can induce a resting state and preclude cells from quitting quiescence and possibly establishing neoplastic lesions.

## Information provided by the tissue architecture and geometry are critical regulators of cell proliferation and quiescence

One of the most important physical properties of an organ is its architecture itself. The normal function of an organ is dependent on its shape and structural features. Indeed, loss of tissue architecture is one of the diagnostic traits of cancer (Nelson and Bissell, 2006). Interestingly, a healthy organ morphology can itself function as a tumor suppressor, suppressing malignancy even in cells possessing several mutations and aneuploidies (Mintz and Illmensee, 1975; Howlett et al., 1995; Weaver et al., 1997, 2002; Wang et al., 2002; Kirshner et al., 2003; Nelson and Bissell, 2006).

Cell quiescence and cytoarchitecture are also exquisitely coupled. The biology of epithelial tissues is a classic example of this relationship. The polarized distribution of cell–cell junctions, organelles, and molecules are the defining morphological traits of epithelial tissues (Inman and Bissell, 2010) and epithelial polarity can have a crucial regulatory effect on cell proliferation (Zeitler et al., 2004). The BM offers a platform of cell anchoring and a source of molecular cues for the correct orientation of apical–basal polarity in epithelia (Weaver et al., 1995; O'Brien et al., 2001; Bissell et al., 2002; Halaoui and McCaffrey, 2015). Reciprocally, cell polarity influences intracellular molecular pathways, providing a mechanism for cells to sense, and assimilate cues from their microenvironment to control metabolism and cell growth pathways and consequently the proliferation-quiescence decision (Jansen et al., 2009; Martin-Belmonte and Perez-Moreno, 2011; McCaffrey and Macara, 2011; Nance and Zallen, 2011; Halaoui and McCaffrey, 2015).

Perturbation of epithelial structure by injury can re-activate the cell cycle. However, epithelial cell–cell interactions induce quiescence when the final organ size is attained (Bryant and Simpson, 1984; Johnston and Gallant, 2002; Zeitler et al., 2004; Zhao et al., 2011), even in the presence of abundant nutrients and growth factors. Nevertheless, loss of cell junctions and polarity is a trait of tumors occurring at the onset and at pre-invasive stages of epithelial cancers (Bissell et al., 2002; Martin-Belmonte and Perez-Moreno, 2011; McCaffrey and Macara, 2011). Several proteins, especially the protein complexes Crumbs, Par, and Scribble, are essential for cell polarity and are deregulated in cancer (reviewed in doi: 10.1038/onc.2014.59 Halaoui and McCaffrey, 2015). In imaginal discs of Drosophila, deletion of *scribble* disrupts epithelial architecture and induces uncontrolled proliferation (Zeitler et al., 2004; Halaoui and McCaffrey, 2015). In mammalian epithelia, depletion of Scrib (a homolog of the Drosophila Scribble) leads to luminal filling due to high rates of proliferation (Zhan et al., 2008) and is sufficient to predispose mice to loss of quiescence control and prostate neoplasia (Pearson et al., 2011). Moreover, loss of the polarity protein Par3 induces mammary tumor growth and metastasis (McCaffrey et al., 2012). Malignant breast cells can be phenotypically reverted from disorganized epithelium to normal-like quiescent acini by inhibiting PI3K signaling. By contrast, PI3K-signaling effectors RAC1 and AKT, respectively, induce epithelial polarity perturbation and unrestrained proliferation via enhanced PI3K activity (Liu et al., 2004). Notably, forcing nuclear actin accumulation in 3D cultures of non-malignant mammary cells resulted in larger and proliferative epithelial structures displaying partially disrupted apical polarity but preserved basal polarity (Fiore et al., 2017). Structures with high levels of nuclear actin had a filled lumen resembling the effects of induced overexpression of ERBb2 or other oncogenes in non-malignant cells (Muthuswamy et al., 2001), which suppress quiescence without perturbing epithelial basal polarity (Spancake et al., 1999; Muthuswamy et al., 2001; Debnath et al., 2002; Liu et al., 2004; Leung and Brugge, 2012; Fiore et al., 2017). These data indicate that acquisition of both basal and apical polarity is required to induce quiescence in epithelial structures (Fiore et al., 2017).

The availability of space within tissues is an important regulator of cell death, quiescence, and proliferation. For instance, cells divide rapidly to fill open spaces and the resultant spatial constraints induce normal cell quiescence maintaining homeostasis (Streichan et al., 2014). Restricting the area available for growth is found to induce cell death, while a wider area increases cell proliferation (Chen et al., 1997). When cultured at high density, cells become quiescent. Tumor cells gradually lose the ability to recognize surrounding tissue architecture and exhibit motility independent of geometrical constraints (Kushiro et al., 2017) such as cell density. But, furthermore, cells residing in tissues with complex anisotropic morphologies have differential access to gradients of growth factors, mitogens, and growth inhibitors, resulting in diverse cell states and fates in different regions of the same tissue (Nelson et al., 2006; Gomez et al., 2010; Hannezo et al., 2017). For instance, Nelson and colleagues showed that tissue geometry dictates concentration gradients of autocrine TGFβ. TGFβ levels were found to be high at the trunk of the microfabricated tubules where cellular quiescence predominated, but were low at the branching/outgrowing tips, resulting in increased invasion and proliferation (Nelson et al., 2006).

It is only in the last two decades that the molecular details of how cells sense density have begun to be unveiled. Several signaling pathways have been implicated in this regulation relaying density signals to induce cell-cycle arrest in response to cell contact (Polyak et al., 1994a; Wieser et al., 1999; Heit et al., 2001; Faust et al., 2005; Zhao et al., 2008; Barry and Camargo, 2013; Gumbiner and Kim, 2014). The Hippo-YAP/TAZ pathway has been found to play important roles in contact inhibition through mechanical cues provided by the microenvironment (Zeng and Hong, 2008; Chen et al., 2012; Halder et al., 2012; Schroeder and Halder, 2012; Gumbiner and Kim, 2014; Mao et al., 2017). Discovered in Drosophila, Hippo-YAP/TAZ signaling is a conserved pathway involved in contact inhibition, mechanotransduction, proliferation, and organ size determination (Piccolo et al., 2014). Alterations in different components of the Hippo pathway have been implicated in cancer (Zeng and Hong, 2008; Zhao et al., 2008; Ma et al., 2014; Piccolo et al., 2014). The Hippo kinases set off a cascade of phosphorylation that culminates in the inactivation of YAP/TAZ, a transcriptional coactivator of cell proliferation and survival genes such as Ki67, c-Myc, Sox4, H19, AFP, BIRC5/survivin, and BIRC2/cIAP1 (Zeng and Hong, 2008; Pan, 2010). The subcellular localization of YAP depends on cell density. YAP is primarily present in the nuclei of cells cultured at low densities, whereas at confluence, YAP is phosphorylated as a consequence of Hippo kinase activity and accumulates in the cytoplasm, where it can no longer act as a transcriptional coactivator (Dong et al., 2007; Zeng and Hong, 2008; Zhao et al., 2010). In addition, formation and stability of adherens junctions and the cadherin–catenin complex in response to cell contact have been shown to stimulate Hippo signaling pathway and induce cell quiescence (Varelas et al., 2010; Schlegelmilch et al., 2011; Barry and Camargo, 2013; Gumbiner and Kim, 2014). Moreover, proteins involved in the regulation of apical–basolateral polarity in epithelia have also been implicated in Hippo-mediated inhibition of YAP/TAZ (Genevet and Tapon, 2011; Boggiano and Fehon, 2012; Richardson and Portela, 2017).

The correct establishment of a quiescent state involves an active process that is controlled by a complex set of signaling cascades including activation of Hippo signaling and attenuation of growth factor stimulated pathways like PI3K-AKT. These are controlled by microenvironmental cues originating from the ECM, tissue architecture, and neighboring cells, and occur despite adequate energy sources and growth factors. In an abnormal microenvironment, these signaling pathways are perturbed resulting in unrestrained cell growth (Figure 2).

## Perspectives and concluding remarks

From the above account, loss of quiescence is a central aspect of tumorigenesis, and it can be seen that the tissue microenvironment plays an essential role in quiescence regulation. Therefore, we argue that in a context-dependent manner the microenvironment can work as either a gas or brake pedal, similar to the analogy proposed to explain the essential roles of oncogenes and tumor suppressors in cell proliferation (Hinds and Weinberg, 1994). A healthy microenvironment may stop cells from re-entering the cell cycle, whereas an aberrant ECM, disruption of tissue and cell architecture, inflammation, and altered metabolism may permit cells to escape quiescence and proliferate uncontrollably. Biochemical and structural properties of the tissue microenvironment and the integration of growth factor and ECM-receptor signaling should be considered when studying cellular quiescence and proliferation and also in cancer diagnostics and treatment.

Looking ahead, it is important for the field of cancer biology to view loss of quiescence as an essential feature of neoplasia and make efforts toward understanding the molecular mechanisms of how quiescence is achieved in normal tissues and how it may be disrupted in cancer. In addition, it is essential to study cellular quiescence in assays that approximate the context that cells experience *in vivo*. Currently, studies designed to identify molecular players in quiescence are based on 2D cell culture models such as contact inhibition, serum deprivation, and cell synchronization that do not include the milieu by which cells are surrounded *in vivo*. Most of these experiments are done by forcing cell cycle synchronization using cytotoxic drugs that affect crucial biochemical pathways such as nucleic acid synthesis and cytoskeleton dynamics during cell division (Spencer et al., 2013). Because these strategies do not reproduce the establishment of quiescence *in vivo* and use strong cell stressors, the biological events of the cell cycle may be masked by assay-intrinsic artifacts. Furthermore, the majority of studies of the cell cycle have been performed by means of bulk assays that conceal the heterogeneous responses displayed by single cells in a population (Spencer et al., 2013). We believe that the appropriate assays for studying the regulation of the proliferation-quiescence decision are those where cells are embedded in 3D and are able to form morpho-functional tissue-like units. For instance, in the mammary acinogenesis assay, non-malignant cells are cultured in a 3D gel of a reconstituted basement membrane and are found to display a program of proliferation and morphogenesis followed by growth arrest and epithelial polarization (Weigelt et al., 2014).

A recent advance in the cell cycle field is the development of live-cell imaging using fluorescent probes for the cycle such as FUCCI and CDK-activity sensors (Spencer et al., 2013; Zielke and Edgar, 2015), which should be considered when planning experiments of quiescence acquisition in physiological contexts. These approaches are overcoming the need for artificially-induced cell cycle arrest and bulk biochemistry, allowing long-term, and real-time tracking of cell cycle dynamics at the single-cell level in asynchronous populations and are, in fact, redefining what we know about the molecular biology of how a cell adopts a proliferative or quiescent state (Spencer et al., 2013; Arora et al., 2017; Barr et al., 2017; Kedziora and Purvis, 2017). Moreover, so far, most attempts to identify molecules involved in the acquisition of quiescence have relied on gene expression profiling methods, such as DNA microarrays and RNA-sequencing, that are unable to discriminate between genes which are a consequence of cell cycle exit and those which play active roles in quiescence induction. The advent of highly robust forward genetic screening strategies such as short-hairpin RNA and CRISPR/Cas9 libraries may greatly contribute to the discovery of intracellular molecules, which relay quiescence-inducing extracellular cues. Indeed, our laboratory is currently performing experiments designed to understand the dynamics and molecular regulation of the proliferation-quiescence decision at the single-cell level in the context of healthy and aberrant tissue microenvironments.

Although essential for tumorigenesis, mutations in proto-oncogenes, and in tumor suppressors and cumulative genetic instability have proved insufficient to explain malignant behaviors, including hyperproliferative phenotypes (Dolberg and Bissell, 1984; Olumi et al., 1999; Bissell and Hines, 2011; Palumbo et al., 2015; Harper et al., 2016; Hosseini et al., 2016). In this review, we showed robust evidence indicating that changes in the cell's surroundings must also occur to affect the proliferation-quiescence homeostasis. Therefore, studies toward understanding cell cycle deregulation in tumor cells should contain aspects of the tissue microenvironment. Furthermore, the development of new therapies to kill fast growing cells in tumors requires an integrative approach taking in account both the cancer cell genetics and the tumor microenvironment.

## Author contributions

AB-C conceived the idea of the review, co-wrote, helped draw the figure panels, and edited the manuscript. AF and PR co-wrote the manuscript and drew the figure panels.

### Conflict of interest statement

The authors declare that the research was conducted in the absence of any commercial or financial relationships that could be construed as a potential conflict of interest.
